# A Triple Band Substrate Integrated Waveguide with Dielectric Resonator Antenna for 4G and 5G Applications

**DOI:** 10.3390/mi14071284

**Published:** 2023-06-22

**Authors:** Irene Kong Cheh Lin, Mohd Haizal Jamaluddin, Abinash Gaya

**Affiliations:** Wireless Communication Centre, School of Electrical Engineering, Universiti Teknologi Malaysia, Johor Bahru 81310, Malaysia; irenekongchehlin@gmail.com (I.K.C.L.); abinashgaya@gmail.com (A.G.)

**Keywords:** SIW, DRA, multiband, 4G, 5G

## Abstract

A triple-band substrate integrated waveguide (SIW) with dielectric resonator antenna (DRA) for fourth-generation (4G) and fifth-generation (5G) applications is proposed and analyzed in this paper. Loading SIW with DRA allows for a wide bandwidth, low losses, and fabrication ease. The proposed antenna can transmit and receive data independently by covering LTE Band 3 at 1.8 GHz, LTE Band 8 at 2.6 GHz, and 5G n77 at 3.7 GHz. A U-shaped cut is applied to achieve the targeted multi-resonance frequencies. The antenna obtains high bandwidths of up to 19.50% with 4.9 dBi gain and 81.0% efficiency at 1.8 GHz, 6.58% bandwidth with 4.4 dBi and 72.7% efficiency at 2.6 GHz, and 8.21% bandwidth with 6.7 dBi and 73.5% efficiency at 3.7 GHz. The simulated and measured results agree well. The proposed antenna is feasible for 4G and 5G applications.

## 1. Introduction

An antenna with a compact size and multiple bands is preferred in this modern era of wireless communication [1,2]. Substrate Integrated Waveguide (SIW), which are planar structures, are developed by connecting the top and bottom metallic ground planes of the dielectric substrate with two periodic rows of metallic vias [3]. It preserves the advantages of conventional classical waveguides in terms of enhanced quality factor, better power handling capability, and compatibility with Printed Circuit Board (PCB) technology [4]. A wide band or dual/multi-band resonance can be achieved by the multiple slots [5] within SIW such as a wideband by a modified dumbbell-shaped slot in [6], a dual-band by two unequal cuts in [5], and a dual-band by an L-shaped cut [7].

Dielectric Resonator Antennas (DRAs) are used extensively in the electromagnetic field in comparison to microstrip antennas because of their superior qualities such as higher radiation efficiency, higher gain, and greater bandwidth [8]. Several research works using the combination of SIW and DRA in a single port have been reported in [9,10,11,12,13]. It can be concluded that a substantial amount of work has been conducted on rectangular DRAs since they provide one extra degree of freedom over cylindrical and two degrees of freedom over hemispherical DRAs [8], resulting in easier bandwidth control. The author in [11] has demonstrated that the loading of DRA in the SIW design structure has improved the impedance bandwidth from 0.9% to 12.4%. Other than that, the impedance bandwidth has increased from 5.0% to 8.8% by changing the rectangular slot to a plus-shaped aperture slot, according to research in [9]. The work in [10] depicts the proposed antenna having a good impedance bandwidth for dual-band frequency for 5G applications. 

In this paper, a rectangular DRA excited by a U-shaped cut in SIW is presented for 4G and 5G applications. The U-shaped cut is -utilized to generate multi resonance frequencies. The simulation of the presented antenna is performed with the help of Ansoft HFSS version 2019. This article is structured as follows: The proposed antenna’s design specifications and the development of the antenna design are covered in Section 2. The results of the simulation and measurement are discussed and shown in Section 3. Major findings are concluded in Section 4.

## 2. Antenna Geometry and Design Consideration

**Figure 1:**
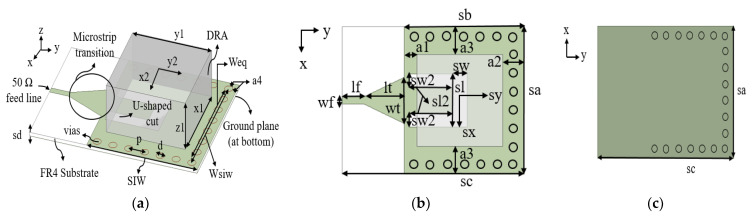
Geometry of the proposed antenna (**a**) 3D view (**b**) Top view (**c**) Back view.

A 3D model view of a single port SIW with a DRA is shown in Figure 1a. A FR4 substrate ($\varepsilon_{r}$ = 4.6) with a loss tangent (tan δ) of 0.019 and a thickness of 1.6 mm is used. Copper with a thickness of 35 μm is cladded on each side of the substrate plane, as depicted in Figure 1b,c. The proposed antenna has an input impedance of 50 Ω and is powered via a microstrip line. The volume of DRA is 40 × 40 × 21 ${mm}^{3}$ with a permittivity of 10 and a loss tangent of 0.0019. The resonant frequency, $f_{0}$ at 1.8 GHz of the dielectric resonator due to the ${TE}_{1\delta 1}^{y}$ can be predicted using the Dielectric Waveguide Model (DWM) based on Equations (1)–(4) [8]:

(1) $$f_{0}=\frac{c}{2\pi \sqrt{\varepsilon_{r}}}\sqrt{k_{x}^{2}{+k}_{y}^{2}{+k}_{z}^{2}}$$

(2) $$k_{x}=\frac{\pi }{x1}$$

(3) $$k_{z}=\frac{\pi }{z1}$$

(4) $$ky\;tan(\frac{ky\;y1}{2})=\sqrt{{(\varepsilon_{r}-1)k}_{0}^{2}{-k}_{y}^{2}}$$

where $k_{x},\;k_{y},\;k_{z}$ are the wavenumbers in x, y, z-direction, $k_{0}$ represents the wave numbers in free space corresponding to the resonant frequency, c is the speed of light in free space, and $\varepsilon_{r}$ is the permittivity of DRA. 

It is excited in its respective mode by the U-shaped cut configuration, which is located in the top wall of SIW and operates in ${TE}_{10}$ mode, with a full ground at the bottom of the substrate. The width, $W_{eq}$ of the effective width of waveguide is calculated from a conventional waveguide WR 430 using Equations (5) and (6) [14]:

(5) $$w_{d}=\frac{w}{\sqrt{\varepsilon_{r}}}$$

(6) $$W_{eq}=w_{d}+\frac{d^{2}}{0.95p}$$

where w is width of the conventional air waveguide, $w_{d}$ is width of dielectric waveguide, $\varepsilon_{r}$ is the permittivity of substrate, d is diameter of the via, and p is separation between each via.

Equation (7) is further refined by including d/$W_{eq}$ ratio because this relation become invalid for larger value of diameter d [14].

(7) $$W_{siw}=W_{eq}-1.08\frac{d^{2}}{p}+0.1\frac{d^{2}}{W_{eq}}$$

The metalized via holes are designed to create SIW. The SIW consists of two rows of metallic vias of diameter, d and separated by a distance, p. To keep energy leakage to a minimal [15], the values of d and p of the vias holes are calculated using Equations (8)–(10) [16]:

(8) $$d<\frac{\lambda_{g}}{5}$$

(9) p≤2d

where guided wavelength, $\lambda_{g}$ is

(10) $$\lambda_{g}=\frac{2\pi }{\sqrt{\frac{{\left(2\pi f\right)}^{2}\varepsilon_{r}}{c^{2}}-{\left(\frac{\pi }{sa}\right)}^{2}}}$$

To avoid any bandgap effects in the operating bandwidth, the following equation should be calculated [15]:

(11) $$\frac{p}{\lambda_{c}}<0.25$$

where $\lambda_{c}$ is the wavelength of cut off frequency.

One end of the SIW is short-circuited to produce standing waves inside the SIW [12]. A microstrip transition (taper) is used to match the impedance between a 50 Ω microstrip line and the SIW. The taper’s initial parameters, lt and wt, are calculated using equations in [17]. The optimal design parameters after the parametric analysis are listed in Table 1.

### 2.1. Evolution of the Design

**Figure 2:**
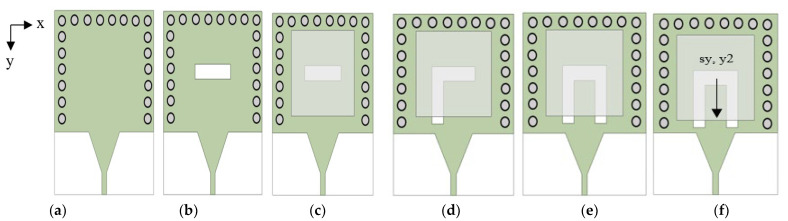
Evolutions of the proposed antenna in top view (**a**) Without cut (**b**) Without DRA (**c**) ANT A—the conventional SIW-DRA antenna (**d**) ANT B—L-shaped cut SIW-DRA antenna (**e**) ANT C—U-shaped cut SIW-DRA antenna (**f**) ANT D—displacement of the DRA and U-shaped cut in *y*-axis.

**Figure 3:**
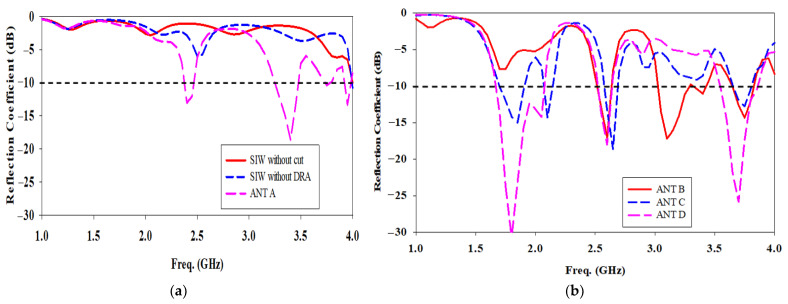
Comparison of the simulated reflection coefficient for evolutions of the proposed antenna (**a**) SIW without cut to ANT A (**b**) ANT B to ANT D.

Six different antenna configurations of the single-port SIW with a DRA are investigated to resonate at 1.8 GHz, 2.6 GHz, and 3.7 GHz to cover the sub-6 GHz frequency for the 5G wireless communication system while also being backwards compatible with 4G bands. The evolutions of the proposed antenna in top view and reflection coefficient are depicted in Figure 2 and Figure 3, respectively. The far end of the SIW is terminated by equally spaced vias to form the feeding cavity [13]. Based on Figure 3a, the resonant frequency is greater than 4 GHz when there is no cut in SIW in Figure 2a. The next steps require the antenna to resonate at the targeted resonant frequencies.

Based on Figure 2b, the SIW with a transverse cut is placed in the centre and in the absence of a DRA is referred to as ‘Without DRA.’ The transverse cut length, sl = $\lambda_{g}/$4 while the width, sw = 0.3 × sl. The cut behaves similar to a magnetic current source. It has been coupled with the electromagnetic energy in the SIW cavity. There is a slight lower return loss at 2.5 GHz in Figure 3a. However, the conventional transverse cut is not enough to couple the electromagnetic energy in the SIW cavity.

The SIW with a transverse cut is then loaded with a DRA and placed in the SIW’s centre, and the antenna is denoted as ‘ANT A’ in Figure 2c. Electromagnetic energy in the SIW cavity is coupled to the DRA element through the cut opening beneath it. The cut behaves similar to a magnetic current source [13], which excites the fields inside the DRA which has a wider bandwidth, greater gain, and higher radiation efficiency. The excited field distributions inside the DR constitute the excited mode, which is associated with a frequency of resonance. The resonant frequencies have been shifted from more than 4 GHz to 2.4 GHz, 3.4 GHz, 3.75 GHz, and 3.95 GHz as shown in Figure 3a. 

As depicted in Figure 2d, a longitudinal cut length, sl2 = $\lambda_{g}/2$ while the width, sw2 = 0.2 × sl2 is added to the end of the left side of the transverse length loaded with the DRA in SIW, forming an L-shaped cut SIW-DRA antenna is known as ‘ANT B’. The L-shaped cut disturbs the surface current density, resulting in a greater radiation in DRA. There is a lower return loss at 1.7 GHz in Figure 3b. However, the electromagnetic energy in the SIW cavity is still not enough to couple to the DRA element at 1.8 GHz through the L-shaped cut opening beneath it. 

In Figure 2e, another identical longitudinal cut length, sl2 and width, sw2 are added to the end of the right side of the transverse length loaded with the DRA in SIW forming a U-shaped cut. SIW-DRA antenna is known as ‘ANT C’. As a result, the most obvious change is that the antenna resonates at 1.85 GHz and 2.1 GHz, but with a narrow bandwidth. 

‘ANT D’ presents the proposed antenna design as shown in Figure 2f. The DRA and U-shaped cut offset positions in the *y*-axis need to be properly chosen as they affect the coupling of energy to the DRA element. Based on Figure 3b, the offset DRA and U-shaped cut improve the bandwidth for the lower and higher frequency bands and shift the middle frequency band to 2.6 GHz by having the best coupling energy to the DRA. As a result, the triple band U-shaped cut SIW fed with a DRA antenna is successfully designed at 1.8 GHz, 2.6 GHz, and 3.7 GHz.

**Figure 4:**
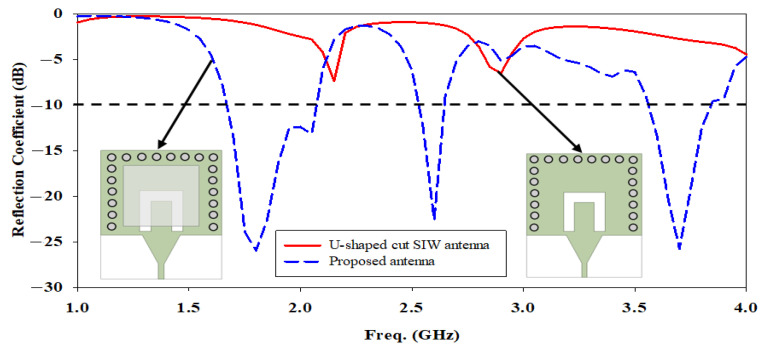
Comparison of the simulated reflection coefficient between the proposed antenna and without DRA.

The reflection coefficient between the U-shaped cut SIW antenna and the proposed antenna has been compared in Figure 4. It clearly shows that the electromagnetic energy in the SIW cavity is optimal to couple to the DRA element through the U-shaped cut opening beneath it, which improves impedance matching by obtaining the widest bandwidth and lowest reflection coefficient at the three aforementioned frequency bands. 

**Figure 5:**
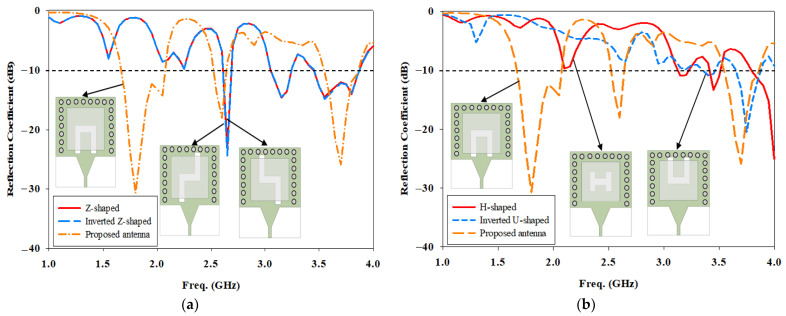
Comparison of the simulated reflection coefficient between U-shaped cut and the other shapes of SIW with DRA antennas (**a**) Z and inverted Z-shaped (**b**) H and inverted U-shaped.

In this work, the reflection coefficient of U-shaped cut is compared to Z-shaped, inverted Z-shaped, H-shaped, and inverted U-shaped is depicted in Figure 5. A U-shaped cut is chosen in SIW fed with a DRA because it gives the best coupling of the energy to the DRA element, achieving resonances at 1.8 GHz, 2.6 GHz, and 3.7 GHz.

### 2.2. Electric Field Distribution

**Figure 6:**
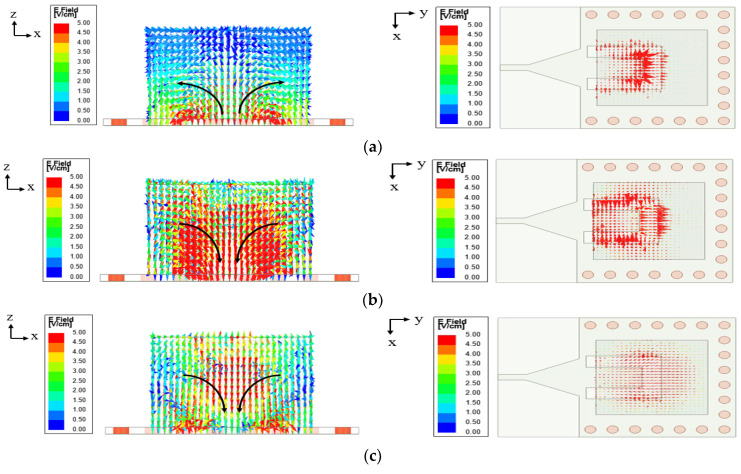
Electric field distribution in the DRA (**a**) ${TE}_{2\delta 1}^{y}$ at 1.8 GHz (from U-shaped cut) (**b**) ${TE}_{2\delta 1}^{y}$ at 2.6 GHz (from U-shaped cut) (**c**) ${TE}_{2\delta 1}^{y}$ at 3.7 GHz (from DRA).

Figure 6 illustrates the electric field distributions in the DRA that are generated at the three mentioned frequencies to determine the resonant frequencies and the DRA’s operating mode. The proposed antenna resonates at 1.8 GHz, as shown in Figure 6a, due to the inner lengths of the U-shaped cut loaded by the DRA, and it induces the DRA’s ${TE}_{2\delta 1}^{y}$ mode. The same ${TE}_{2\delta 1}^{y}$ mode of DRA is also induced at 2.6 GHz, but in different directions, as shown in Figure 6b, and it is caused by the outer lengths of the U-shaped cut loaded by the DRA. As shown in Figure 6c, the DRA radiates at 3.7 GHz with ${TE}_{2\delta 1}^{y}$ mode while acting as a medium for radiation for the U-shaped cut at 1.8 GHz and 2.6 GHz.

### 2.3. Parametric Studies

In this section, the influence of design parameters on the bandwidth response of the proposed antenna has been carried out. Parametric studies are conducted for optimal parameter selection. 

**Figure 7:**
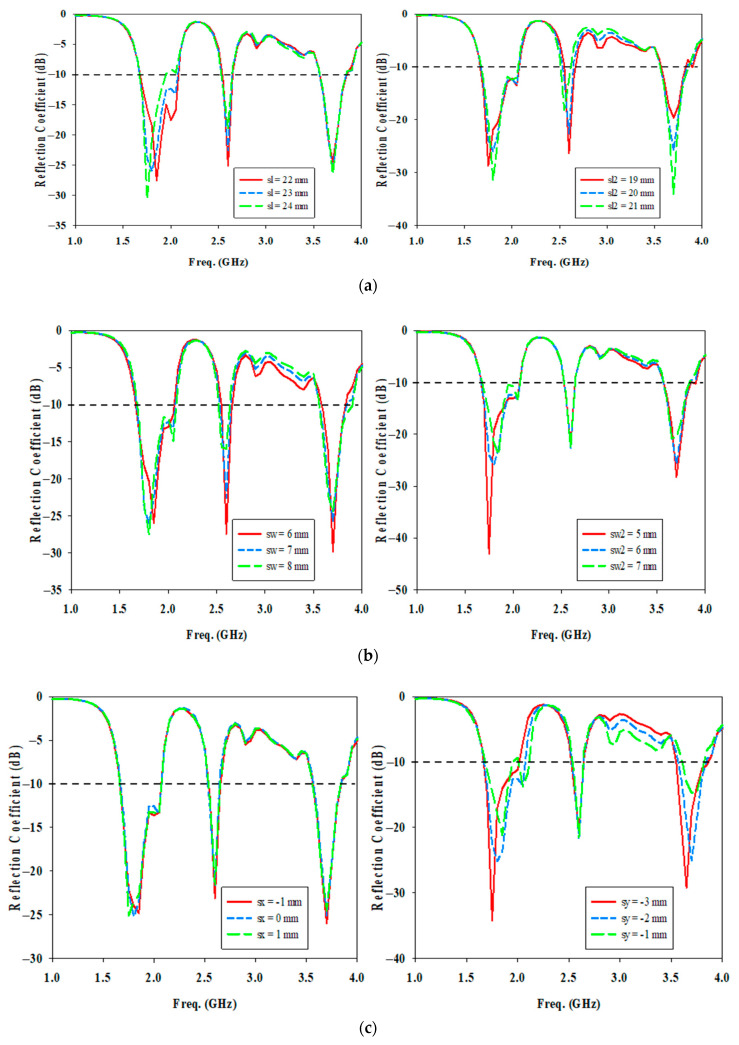
Simulated reflection coefficient for different U-shaped cut parameters (**a**) cut length (**b**) cut width (**c**) cut position.

Firstly, the effects of U-shaped cut size and its position are studied to determine the best coupling to the DRA. Figure 7a shows the variations in the cut length for sl from 22 mm to 24 mm and sl2 from 19 mm to 21 mm, with a step size of 1 mm. By increasing sl, the resonance frequency of the lower frequency band (1.8 GHz) is affected only, and it is shifted towards the lower band. The optimum value of sl = 23 mm gives the required resonance frequency and the widest impedance bandwidth at 1.8 GHz. An increase in sl2 length introduces a mismatch in the reflection coefficient for all three frequencies as well as a shift in resonant frequency at the first and middle frequency ranges. When sl2 = 20 mm, it provides the best coupling of energy to the DRA element.

Figure 7b show the variations in the cut width for sw from 6 mm to 8 mm and sw2 from 5 mm to 7 mm, with a step size of 1 mm. A larger variation in the cut width causes change in the magnitude of the reflection coefficient as well as shift in resonant frequency in the lower and middle frequency ranges. An increase of sw2 shifts the resonance frequency of lower frequency band to higher and higher frequency band to lower.

Based on Figure 7c, it is quite apparent that the cut position in *x*-axis, sx has a negligible effect on the bandwidth at the three frequency bands. The only difference is the shift of resonant frequency at lower frequency band when sx increases. When sx = 0 mm, the antenna resonates at 1.8 GHz sharply where the maximum electric field flows through ${TE}_{10}$ of SIW which is designed at 1.8 GHz, therefore it is chosen. Change in cut position in *y*-axis, sy affects the reflection coefficients at the lower and upper resonant frequencies only. A decrease of sy shifts both resonance frequency of lower and higher frequency bands to lower. When the U-shaped cut is not too close to the microstrip transition where sy = −2 mm, it has the widest impedance bandwidth at 1.8 GHz and is resonating at 1.8 GHz and 3.7 GHz.

**Figure 8:**
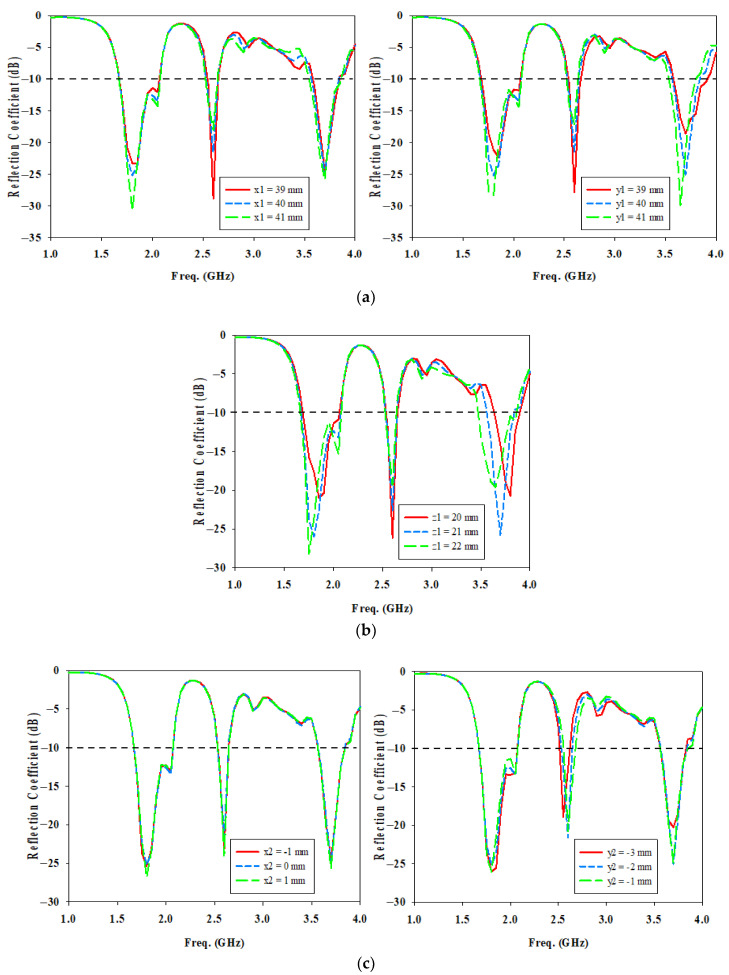
Simulated reflection coefficient for different DRA parameters (**a**) DRA length and width (**b**) DRA height (**c**) DRA position.

The length and width of the DRA, denoted as x1 and y1 in Figure 8a, are another factor that controls the resonance frequency shift. As x1 increases, it introduces a mismatch in the bandwidth at the middle and higher frequency ranges. This is due to the fact that the electromagnetic energy in the SIW cavity is not optimal to couple to the DRA element at 2.6 GHz and 3.7 GHz through the U-shaped cut opening beneath it. As yl increases, it shifts the resonant frequency in the lower and higher frequency ranges to lower because of the increase in the effective width of the DRA element in the y-direction, which is parallel to the feeding port. This results in a decrease in the wave number in the y-direction (ky), resulting in the resonance frequency getting smaller.

Figure 8b clearly indicates that as the height of the DRA, z1 increases, the resonance frequency of both 1.8 GHz and 3.7 GHz shifts towards the lower band because of the increase in the effective height of the DRA element in the z-direction. This results in a decrease in the wave number in the z-direction (kz), resulting in the resonance frequency getting smaller.

Based on Figure 8c, it is quite apparent that the DRA position in the *x*-axis, x2 has a negligible effect on the reflection coefficient at the three frequency bands. However, a change in DRA position in *y*-axis, y2 affects the reflection coefficients at the middle resonant frequency. When y2 = −2 mm which is closer to the microstrip transition, it gives the best coupling of the energy for the U-shaped cut to the DRA element at 2.6 GHz. 

## 3. Results and Discussion

**Figure 9:**
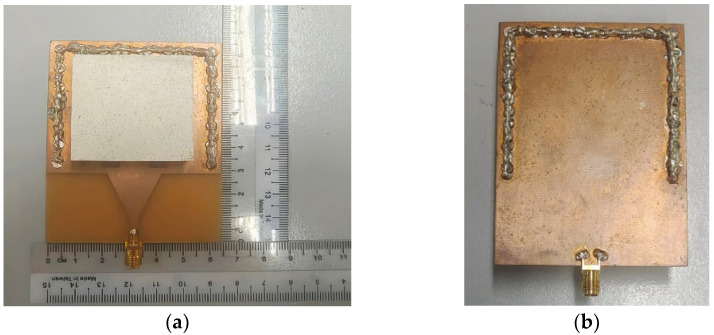
Prototype of the single port SIW with a DRA (**a**) Top view (**b**) Back view.

The top and bottom views of the prototype of the single port SIW with a DRA are shown in Figure 9a,b, respectively. The DR is made of ECCOSTOCK HiK dielectric material with a permittivity of 10, while the microstrip feeding line and ground plane are etched on a FR4 substrate with a permittivity of 4.6. The holes for the vias have been drilled and metalized. The prototype is excited by a female 50 Ω SMA connector.

**Figure 10:**
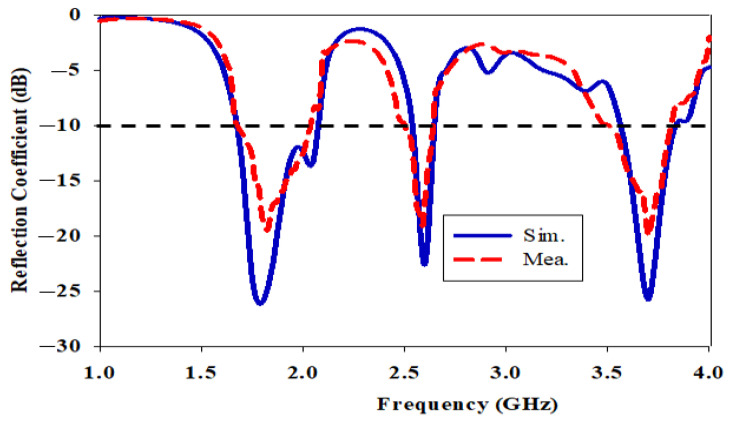
Simulated and measured reflection coefficient of the single port SIW with a DRA.

Figure 10 shows a comparison of the simulated and measured reflection coefficients using an impedance bandwidth reference level of −10 dB. As indicated in Figure 10, the prototype matches well at all three frequencies. At 1.8 GHz, the simulated and measured impedance bandwidths are 21.51% (1.670–2.070 GHz) and 19.50% (1.675–2.035 GHz) at 1.8 GHz, whereas they are 5.78% (2.520–2.670 GHz) and 6.58% (2.500–2.670 GHz) at 2.6 GHz. At 3.7 GHz, they are 7.57% (3.560–3.840 GHz) and 8.21% (3.505–3.805 GHz). Based on Figure 10, there is a slight difference in agreement between the measured results and the simulated results of the fabricated antenna. This is because imperfect fabrication and the adhesive glue that is used to stick the DRA to the substrate plane cause the air gap. The DRA does not radiate well, resulting in not achieving the full bandwidth. Table 2 tabulates each simulated and measured antenna parameter. Due to connector loss and imperfect fabrication, there is a slight reduction in gain in the measurement. The measured total efficiency is lower than the simulated one due to the lower gain and greater return loss in the measurement compared to the simulation.

**Figure 11:**
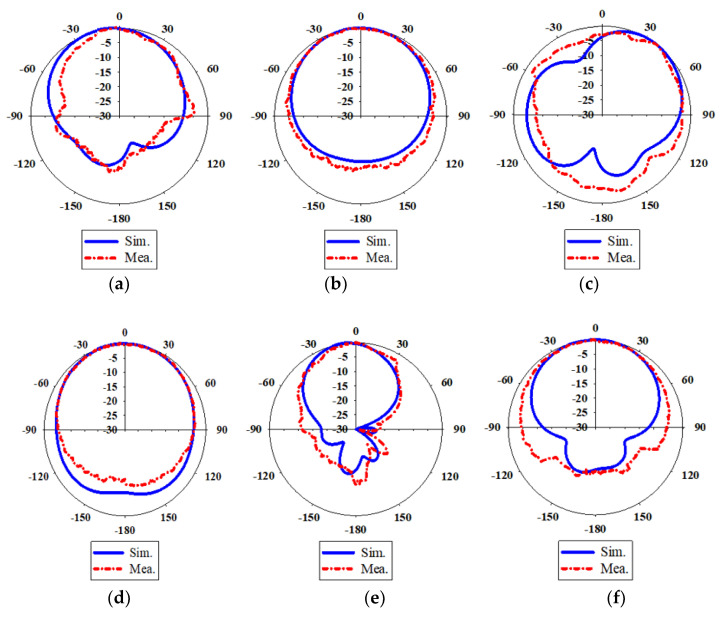
Normalized simulated and measured radiation patterns of the single port SIW with a DRA (**a**) E-Plane at 1.8 GHz (**b**) H-Plane at 1.8 GHz (**c**) E-Plane at 2.6 GHz (**d**) H-Plane at 2.6 GHz (**e**) E-Plane at 3.7 GHz (**f**) H-Plane at 3.7 GHz.

All simulated radiation patterns point in broadside directions for all three frequencies except the E-plane radiation pattern at 2.6 GHz, which points at 45°, as shown in Figure 11c. This is due to the modification of the conventional horizontal cut in SIW, which changes the peak of the radiation pattern. The resonances at 1.8 and 2.6 GHz are caused by the U-shaped cut. The gain at 1.8 GHz is 5.0 dBi. However, the gain at 2.6 GHz is lower, that is, 4.5 dBi, and the beamwidth at 2.6 GHz is the largest because strong electric fields come from the top and bottom to the center of the U-shaped cut in SIW, causing a concentration of electric fields inside the inner of the U-shaped cut and cancellation of top and bottom electric fields in the xy plane, resulting in a reduction of gain and a smaller gain, as shown in Figure 6b. Due to the radiating of DRA, the frequency of 3.7 GHz has a directional radiation pattern in both the E-field and the H-field. As a result of the DRA resonance, a higher gain of 6.9 dBi is obtained. The beamwidth at 3.7 GHz is the narrowest when the highest gain is obtained. Figure 11 shows the E-plane and H-plane views of the normalized simulated and measured radiation patterns in the sequence of described frequencies. As indicated in Figure 11, there are some differences in the simulated and measured radiation patterns. These radiation patterns have deteriorated due to the disturbances of imperfect fabrication and connectors.

Table 3 compares antenna parameters in terms of bandwidth, gain, and total antenna efficiency in single port SIW designs using the proposed antenna and current structures. Based on the outcomes shown in Table 3, changing the shape of the slots/cuts and merging with DRA results in a wider bandwidth and various resonances. Firstly, when compared to the broadband antenna in [6], the proposed antenna delivers multiple resonance benefits. Furthermore, it has a wider bandwidth performance and the previously reported benefit over the single band antennas in [18,19], and the dual-band antennas in [5,7]. The proposed antenna that combines SIW and a DRA outperforms the same U-shaped slot/cut SIW antenna in [20] in terms of bandwidth performance and multiple resonances. Moreover, for SIW and DRA work, the proposed triple band SIW with a DRA has more selections of frequency switching and a wider bandwidth than the single band antennas in [9,11,12] and a dual-band antenna in [10]. The proposed single port triple band SIW with a DRA antenna is backward compatible with 4G bands and also supports the sub-6 GHz band for future 5G wireless communication systems.

## 4. Conclusions

A multiband SIW with a single-element DRA is designed, fabricated, and tested. The U-shaped cut is used to enable multi-band operation at frequencies of 1.8 GHz, 2.6 GHz, and 3.7 GHz while providing a compact antenna size. The proposed antenna demonstrates a high bandwidth and a good gain at each frequency band. The simulated and measured results agree well. This research can be extended by applying Multiple-Input Multiple-Output (MIMO) technology by utilizing multiple antennas at the transmitting and receiving ends of the communication system [21] to combat multipath fading, resulting in increased channel capacity and spectral efficiency This is a promising approach to meet the demand for large data rates [22]. 

## Figures and Tables

**Table 1:** Optimized antenna dimensions.

Parameter	Value (mm)	Parameter	Value (mm)	Parameter	Value (mm)	Parameter	Value (mm)
sa	64	x2	0	sw	7	a1	6
sb	56	y2	−2	sl2	20	a2	10
sc	85	lf	10	sw2	6	a3	12
sd	1.6	lt	19	sx	0	a4	2
x1	40	wf	2.95	sy	−2	W_eq_	52
y1	40	wt	20	d	4	W_siw_	56
z1	21	sl	23	p	8		

**Table 2:** Comparison between simulation and measurement of each antenna parameter for the single port SIW with a DRA.

Antenna Parameters		1.8 GHz	2.6 GHz	3.7 GHz
Covered Bandwidth (%)	Sim.	21.51	5.78	7.57
	Mea.	19.50	6.58	8.21
Gain (dBi)	Sim.	5.0	4.5	6.9
	Mea.	4.9	4.4	6.7
Total Antenna Efficiency (%)	Sim.	83.9	74.4	76.9
	Mea.	81.0	72.7	73.5

**Table 3:** Comparison of proposed structure with another existing antenna.

Year [Ref.]	Shapes of Slot/Cut	DRA	Frequency (GHz)	Bandwidth (%)	Gain (dBi)	Total Antenna Efficiency (%)
2014 [18]	A horizontal slot	No	2.60	16.05	5.1	92.0
2013 [19]	Two vertical cuts	No	3.60	12.61	Not stated	Not stated
2019 [6]	A dumbbell-shaped slot	No	22.00	26.06	8.0	Not stated
2019 [20]	A U-shaped slot	No	28.00	13.79	7.2	Not stated
2013 [7]	L-shaped slot	No	10.93 12.69	1.56 1.42	Not stated	Not stated
2015 [5]	Two vertical cuts	No	28.00 38.00	1.61 5.82	5.2 5.9	Not stated
2020 [11]	A horizontal cut	1 RDRA	5.90	12.37	4.7	Not stated
2016 [9]	A plus-shaped cut	1 CDRA	6.56	8.54	3.7	Not stated
2014 [12]	A H-shaped slot	2 RDRAs	60.00	10.03	5.5	81.0
2021 [10]	A horizontal cut	1 RDRA	28.00 38.00	13.62 9.94	Not stated	Not stated
Current proposed design	A U-shaped cut	1 RDRA	1.80 2.60 3.70	19.50 6.58 8.21	4.9 4.4 6.7	81.0 72.7 73.5

## Data Availability

Data is contained within the article.
